# Efficacy, Effectiveness, and Immunogenicity of the Respiratory Syncytial Virus Prefusion F Vaccine Administered During Pregnancy: Protocol for a Living Systematic Review and Meta-Analysis

**DOI:** 10.2196/94382

**Published:** 2026-10-15

**Authors:** Agustina Mazzoni, Ariel Bardach, Mabel Berrueta, Agustin Ciapponi, Karen Klein, Martin Brizuela, Carolina Nigri, Noelia Castellana, Jamile Ballivian, Juan Manuel Sambade, Katharina Stegelmann, Divya Patel, Jessica E Atwell, Jennifer Deese, Sarah N Cox, Lu Wang, Xu Xiong, Andy Stergachis, Flor M Munoz, Pierre Buekens

**Affiliations:** 1 Institute for Clinical Effectiveness and Health Policy (IECS), Lavalleja 856, Buenos Aires, C1414DTR, Argentina, 54 0111558570687; 2 Medical Evidence Development, Research & Development, Pfizer, Inc., New York, NY, United States; 3 Maternal Immunization Team, Global Medical Affairs, Pfizer (United States), New York, NY, United States; 4 Statistical Research and Data Science Center, Data Sciences and Analytics, Pfizer, Inc., New York, NY, United States; 5 Department of Epidemiology, Celia Scott Weatherhead School of Public Health, Tulane University, New Orleans, LA, United States; 6 Departments of Pharmacy and Global Health, University of Washington, Seattle, WA, United States; 7 Departments of Pediatrics and Molecular Virology and Microbiology, Baylor College of Medicine, Houston, TX, United States

**Keywords:** respiratory syncytial virus vaccine, RSV vaccine, effectiveness, immunogenicity, pregnancy, living systematic review

## Abstract

**Background:**

Respiratory syncytial virus (RSV) is a leading cause of lower respiratory tract disease in infants, with the highest burden in the first 6 months of life. Maternal immunization with the bivalent (RSV-A and RSV-B) prefusion F (RSVPreF) protein subunit vaccine (ABRYSVO) is a recently recommended strategy for preventing RSV-related outcomes in infants. First licensed for use in pregnancy in 2023, the RSVPreF vaccine demonstrated efficacy in a single pivotal randomized trial (Maternal Immunization Study for Safety and Efficacy; MATISSE); postlicensure real-world evidence on vaccine effectiveness across settings, seasons, and subgroups and on antibody durability and transplacental transfer remains limited and has not been synthesized in a continuously updated framework, motivating this review.

**Objective:**

This living systematic review and meta-analysis will synthesize emerging postlicensure data on the efficacy, effectiveness, and immunogenicity of the RSVPreF vaccine in real-world settings.

**Methods:**

We will conduct, in real time, a living systematic review and meta-analysis following Cochrane methods and PRISMA-P (Preferred Reporting Items for Systematic Reviews and Meta-Analyses Protocols) guidance, including randomized clinical trials and postlicensure real-world studies (eg, cohort, case-control, test-negative, and quasi-experimental studies). Primary objectives are evaluation of vaccine efficacy and effectiveness in preventing RSV-specific and all-cause respiratory disease and immunogenicity in infants and pregnant individuals. Vaccine effectiveness outcomes include RSV laboratory-confirmed infection, RSV acute respiratory illness, RSV lower respiratory tract disease, RSV hospitalization, RSV complications, all-cause lower respiratory tract disease and hospitalization, and antibiotic use in infants with RSV infection. Immunogenicity outcomes include maternal neutralizing antibody titers, maternal-infant transfer ratios, infant titers over time, and RSV viral load. We will search MEDLINE, Embase, the Cochrane Library, LILACS, Science Citation Index Expanded, Chinese biomedical and citation databases, clinical trial registries from January 2023 onward with no language restrictions, updating at least monthly. Two investigators will independently select studies, extract data, and assess risk of bias. Adjusted estimates will be standardized to vaccine efficacy and effectiveness. Certainty will be graded using the Grading of Recommendations Assessment, Development, and Evaluation (GRADE) system.

**Results:**

This living systematic review was funded in June 2025. Searches began in January 2023 and will continue with surveillance updates at least monthly until April 2028. As of January 2026, 837 records were screened, 105 (12.5%) were assessed in full text, and 12 (1.4%) met the inclusion criteria, of which 10 (1.2%) contributed extractable data. Data collection and analysis are ongoing and displayed on an interactive dashboard. Findings will be disseminated through peer-reviewed publications, conferences, and a real-time dashboard, with the first effectiveness estimates available in July 2026.

**Conclusions:**

This living systematic review and meta-analysis will provide a continuously updated synthesis of real-world evidence on maternal RSVPreF vaccination to inform clinical and policy decisions.

## Introduction

Worldwide, respiratory syncytial virus (RSV) contributes substantially to infant morbidity and mortality, accounting for tens of millions of lower respiratory tract disease (LRTD) cases and approximately 100,000 deaths among children under 5 years of age each year, with the highest risk concentrated in the first 6 months of life and in low- and middle-income countries [1,2]. Approximately half of hospitalizations and deaths due to RSV LRTD occur in infants under 6 months of age, with an estimated 6.6 million episodes of LRTD, a hospitalization rate ranging from 15 to 20 per 1000 infants, and an intensive care unit admission rate of 10% to 15% of hospitalized cases. The associated morbidity of the infection can affect children for up to 2 years, and possibly longer, after the first episode, with an increase in health care use and medication prescription [1,2].

Maternal immunization offers a pragmatic strategy to bridge this early-life vulnerability via transplacental transfer of neutralizing antibodies. In 2023, the bivalent RSV prefusion F (RSVPreF) protein subunit vaccine (ABRYSVO) became the first and only vaccine licensed for maternal use to protect infants against both RSV-A and RSV-B from birth to 6 months of age in multiple jurisdictions, with approvals and recommendations subsequently issued by major agencies (eg, the Food and Drug Administration and Centers for Disease Control and Prevention in the United States and the European Medicines Agency and UK Joint Committee on Vaccination and Immunisation) [3,4]. The World Health Organization (WHO) recommends implementation of maternal RSVPreF and/or monoclonal antibody therapy based on country-specific considerations, including cost-effectiveness, financing capacity, supply chain feasibility, anticipated coverage, and health system integration capabilities. Countries may consider dual implementation strategies incorporating both maternal vaccination and infant monoclonal antibody prophylaxis, although such comprehensive approaches present additional programmatic complexity and operational challenges [5].

A pivotal phase 3 clinical trial (Maternal Immunization Study for Safety and Efficacy; MATISSE) evaluated the efficacy of the RSVPreF vaccine administered to pregnant individuals between 24 and 36 weeks of gestational age and showed an efficacy of 82.4% (95% CI 57.5%-93.9%) against medically attended RSV LRTD among infants within 90 days after birth and 70.0% (95% CI 50.6%-82.5%) within 180 days after birth. The efficacy against hospitalization associated with RSV infection was 69.7% (95% CI 37.1%-86.7%) within 90 days of birth and 55.3% (95% CI 23.8%-74.6%) within 180 days of birth [6,7]. As vaccine rollout progresses, high-quality real-world evidence (RWE)—drawn from electronic health records, administrative databases, surveillance systems, and registries—will be indispensable to quantify effectiveness across settings, seasons, and subgroups; understand the role of vaccination timing relative to delivery; and evaluate the durability of transferred antibodies.

To date, 3 systematic reviews have evaluated the efficacy of RSV vaccination during pregnancy to prevent outcomes associated with RSV infection and/or disease. Vaccine efficacy (VE) for preventing RSV LRTD ranged from 54% to 57.3%, from 74% to 81.9% for RSV severe LRTD, and from 54% to 67.9% for RSV LRTD hospitalization in infants under 3 months of age [8,9]. Another review estimated a relative risk of 0.50 (95% CI 0.31-0.82) for preventing RSV laboratory-confirmed hospitalization in the same population [10]. These reviews, however, were based predominantly on prelicensure randomized trials and, therefore, reflect efficacy under trial conditions rather than real-world effectiveness of the licensed maternal RSVPreF vaccine. They provide limited information on policy-relevant subgroups (such as the interval from vaccination to delivery and gestational age at vaccination); do not integrate maternal and postpartum outcomes or immunogenicity; and, as they are static one-time syntheses, become outdated rapidly as postlicensure RWE accumulates. A continuously updated, real-world synthesis specific to maternal RSVPreF is therefore needed to fill this gap.

This protocol outlines a living systematic review and meta-analysis [11] focused on the efficacy, effectiveness, and immunogenicity of maternal RSVPreF in infants and pregnant and postpartum individuals. We emphasize rigorous inclusion of RWE and transparent, design-appropriate synthesis to inform clinical and policy decisions in real time. Safety outcomes are beyond the scope of this review and will be addressed separately. We aim to estimate the efficacy and effectiveness of maternal RSVPreF vaccination in preventing RSV laboratory-confirmed infection, RSV acute respiratory illness, RSV LRTD, RSV hospitalization, RSV complications (including secondary bacterial infection, respiratory failure, multi-organ failure, and death), all-cause LRTD, all-cause LRTD hospitalization, and antibiotic use in infants with RSV infection and characterize vaccine-induced immune responses in pregnant persons and infants. Immunogenicity outcomes are included as secondary outcomes to complement clinical efficacy and effectiveness estimates. Specifically, they assess maternal neutralizing antibody titers and maternal-to-infant transfer ratios as potential correlates of protection, provide mechanistic insights into the influence of gestational age at vaccination and the interval between vaccination and delivery, and help characterize the waning of infant protection over time. These outcomes may also support inference in subgroups or settings where clinical outcome data remain limited. We also aim, in secondary objectives, to evaluate effect modification by gestational timing of vaccination; interval from vaccination to delivery; gestational age at birth; infant risk factors; RSV subtype; coadministration with other pregnancy vaccines; and country income level, region, and seasonal pattern, as well as quantifying maternal-infant antibody transfer ratios and assessing antibody kinetics in infants. The results of this living review will provide health care professionals, policymakers, and researchers with robust, up-to-date evidence to guide real-world use of the RSVPreF vaccine during pregnancy.

## Methods

### Design

We will conduct a living systematic review and meta-analysis according to the Cochrane methods [12] and PRISMA-P (Preferred Reporting Items for Systematic Reviews and Meta-Analyses Protocols) guidelines [13]. The protocol is registered in PROSPERO [14] under registration CRD420251077829. Scheduled surveillance updates will occur at least monthly, with rapid updates during peak publication periods.

### Eligibility Criteria

The population consists of pregnant persons, postpartum persons, and infants born at any gestational age, including preterm infants and those admitted to neonatal intensive care. Infant age will be defined chronologically from live birth to 12 months. Gestational age at birth will be extracted and classified as term (≥37 weeks) or preterm (<37 weeks), with subcategories used where reported. Results based on corrected age will be identified and considered separately.

We will include studies reporting efficacy or effectiveness outcomes with sample sizes of at least 50 participants and immunogenicity outcomes with at least 10 participants. The intervention to be evaluated is the RSVPreF vaccine (ABRYSVO) administered during pregnancy within nationally licensed gestational windows as a single 0.5-mL intramuscular dose after reconstitution. The primary effectiveness comparison will be maternal RSVPreF vaccination vs no maternal RSVPreF vaccination. For infant outcomes, comparison groups will comprise infants born to pregnant individuals who did not receive the RSVPreF vaccine; for maternal outcomes, comparison groups will comprise unvaccinated pregnant or postpartum individuals. Placebo-controlled trials, if eligible, will be synthesized separately from observational studies. Alternative preventive products, including infant nirsevimab, will not be pooled with inactive comparators; direct active comparator evidence will be analyzed as a separate comparative effectiveness question. Infant RSV monoclonal prophylaxis will be extracted as a potential cointervention where appropriate. In test-negative studies, cases and controls will be defined by RSV test status, whereas maternal vaccination status will define the exposure comparison.

Study designs will include randomized controlled trials (RCTs; phase 2 and 3) and real-world postlicensure observational studies (eg, cohort, case-control, test-negative, or quasi-experimental studies). Systematic reviews will be used only as a source to identify primary studies. Case series will be described narratively and excluded from quantitative synthesis. Animal studies will be excluded.

### Information Sources, Study Selection, and Data Management

We will search MEDLINE (PubMed), Embase (Ovid), Cochrane Library, LILACS, Science Citation Index Expanded, China National Knowledge Infrastructure, the Chinese Biomedical Literature Database, the Chinese Science Citation Database, ClinicalTrials.gov, the European Union Clinical Trials Register, the WHO International Clinical Trials Registry Platform, and gray literature from public health agencies (the Centers for Disease Control and Prevention, UK Health Security Agency, UK Joint Committee on Vaccination and Immunisation, and WHO; Multimedia Appendix 1). Coverage will encompass January 2023 onward (postlicensure era) up to April 2028, with no language restrictions. A peer-reviewed strategy will combine controlled vocabulary and text words for RSV, RSVPreF and ABRYSVO, pregnancy, and immunogenicity; test-negative and other RWE designs will be explicitly captured. Hand searching of references and expert consultation will complement electronic searches. Pairs of reviewers will independently screen titles and abstracts and full texts using the Nested Knowledge platform [15], extract data via piloted and validated REDCap forms (Vanderbilt University) [16], and resolve disagreements through consensus or third-party adjudication. All screening will be performed by human reviewers; no machine learning or automated classification tool will be used for study selection. Extracted data will include study setting, design, population, vaccination details (gestational age, interval to delivery, and coadministration), outcomes and definitions, effect measures (adjusted and unadjusted), follow-up windows, and funding and conflicts. Standardized procedures, including peer verification and automated consistency checks, will ensure data quality and traceability. The living systematic review will maintain a public changelog documenting updates, versioning, and any resolved discrepancies.

### Definitions and Outcome Ascertainment in Vaccinated Populations

Efficacy and effectiveness outcomes include RSV laboratory-confirmed infection, RSV acute respiratory illness, RSV LRTD, RSV hospitalization, RSV complications (including secondary bacterial infection, respiratory failure, multi-organ failure, and death), all-cause LRTD, all-cause LRTD hospitalization, and antibiotic use in infants with RSV infection (Table 1 and Multimedia Appendix 2). We will prioritize laboratory confirmation of RSV via nucleic acid amplification testing (eg, real-time polymerase chain reaction) or validated antigen assays for case ascertainment in both infants and mothers. Immunogenicity outcomes will include quality and quantity of humoral and cellular immune responses and RSV viral load (Table 2).

**Table 1.** Efficacy and effectiveness outcomes and definitions^a^.

Outcome family	Population	Episode measure	Operational definition
RSV^b^-specific outcomes			
Any RSV detection	Infants and pregnant and postpartum individuals	First and any	Laboratory-confirmed RSV detection irrespective of symptoms
Asymptomatic RSV infection	Infants and pregnant and postpartum individuals	First and any	Laboratory-confirmed RSV infection in the absence of symptoms
RSV ARI^c^	Infants and pregnant and postpartum individuals	First and any	Clinical disease consistent with RSV ARI based on laboratory confirmation and/or relevant diagnostic codes
RSV LRTI^d^ or LRTD^e^	Infants and pregnant and postpartum individuals	First and any	Clinical disease consistent with RSV LRTI or LRTD based on laboratory confirmation and/or relevant diagnostic codes
RSV hospitalization	Infants and pregnant and postpartum individuals	First and any	Hospital admission due to laboratory-confirmed RSV illness
RSV LRTI or LRTD hospitalization	Infants and pregnant and postpartum individuals	First and any	Hospital admission due to clinical disease consistent with RSV LRTI or LRTD
RSV-related complications	Infants	First and any	Secondary bacterial infection, respiratory failure, multi-organ failure, or death following RSV ARI, LRTI, or LRTD
All-cause respiratory outcomes			
All-cause ARI	Infants and pregnant and postpartum individuals	First and any	Clinical disease consistent with ARI irrespective of RSV attribution
All-cause LRTI or LRTD	Infants and pregnant and postpartum individuals	First and any	Clinical disease consistent with LRTI or LRTD irrespective of RSV attribution
All-cause LRTI or LRTD hospitalization	Infants and pregnant and postpartum individuals	First and any	Hospital admission due to clinical disease consistent with LRTI or LRTD
Other outcomes			
Antibiotic use with RSV infection	Infants	First and any	Antibiotic use among infants with RSV infection

a Infants were assessed from birth to 12 months of age. Pregnant and postpartum individuals were assessed up to 12 months after respiratory syncytial virus prefusion F vaccination. First and any episodes will be extracted separately where applicable. For complications, first and any episodes apply to secondary bacterial infection, respiratory failure, and multi-organ failure; death is recorded as a single event.

b RSV: respiratory syncytial virus.

c ARI: acute respiratory illness.

d LRTI: lower respiratory tract infection.

e LRTD: lower respiratory tract disease.

**Table 2.** Immunogenicity outcomes and measurements.

Outcome and population	Variable operational definition
Humoral immune response quantity	
Infants	Antibody measurements (IgM^a^, IgG^b^, IgA^c^, IgM, IgG, IgA, and combined IgM/IgG; neutralizing antibodies; and transplacental transfer ratios) in infant umbilical cord blood at or near delivery or peripheral blood at or near delivery with known sample timing relative to RSVPreF^d^ vaccination
Pregnant and postpartum individuals	Antibody measurements (IgM, IgG, IgA, IgM, and IgG combined and neutralizing antibodies), maternal serum at or near delivery, or maternal breast milk with known sample timing relative to RSVPreF vaccination
Cellular immune response quantity	
Infants	Cellular measurements (T_h_1 [IFN-γ^e^–expressing T cells], T_h_2 [IL-4^f^–expressing T cells], balanced T_h_1 and T_h_2, CMI^g^, T-cell responses, ELISpot^h^ data, and intracellular cytokines) in infant umbilical cord blood at or near delivery or peripheral blood at or near delivery with known sample timing relative to RSVPreF vaccination
Pregnant and postpartum individuals	Cellular measurements (T_h_1 [IFN-γ–expressing T cells], T_h_2 [IL-4–expressing T cells], balanced T_h_1 and T_h_2, CMI, T-cell responses, ELISPOT data, and intracellular cytokines) in maternal serum at or near delivery or maternal breast milk with known sample timing relative to RSVPreF vaccination
RSV^i^ viral load	
Infants	Viral copy measurement present in infant blood or respiratory samples at time of illness
Pregnant and postpartum individuals	Viral copies present in maternal blood or respiratory samples at time of illness

a IgM: immunoglobulin M.

b IgG: immunoglobulin G.

c IgA: immunoglobulin A.

d RSVPreF: respiratory syncytial virus prefusion F.

e IFN-γ: interferon gamma.

f IL-4: interleukin-4.

g CMI: cell-mediated immunity.

h ELISpot: enzyme-linked immunosorbent spot.

i RSV: respiratory syncytial virus.

### Risk of Bias in Individual Studies

We will assess risk of bias using version 2 of the Cochrane risk-of-bias tool for randomized trials (RoB 2) [17] and the Risk of Bias in Nonrandomized Studies of Interventions (ROBINS-I) for nonrandomized comparative studies [18]. National Institutes of Health quality assessment tools will be used for noncomparative studies [19]. Two reviewers will perform independent assessments with consensus resolution.

### Data Synthesis and Statistical Analysis

We will conduct design-specific meta-analyses (eg, separate pools for RCTs vs observational studies) using random-effects models for efficacy and effectiveness outcomes and for selected immunogenicity outcomes. Continuous immunogenicity outcomes will not be quantitatively pooled due to the anticipated heterogeneity in laboratory assays, measurement scales, and reporting formats across studies. Rather, these outcomes will be summarized descriptively using the values reported in the original studies. Random-effects models were selected a priori independent of the magnitude of statistical heterogeneity due to the anticipated clinical and methodological diversity across included studies, including differences in study design, settings, RSV seasonality, gestational timing of vaccination, and interval between vaccination and delivery.

For analyses including a comparator group, random-effects models will be performed using inverse variance weighting to calculate pooled effect estimates with corresponding 95% CIs. Between-study variance (τ^2^) will be estimated using the restricted maximum likelihood method. CIs for pooled estimates will be calculated using the Hartung-Knapp-Sidik-Jonkman adjustment based on the *t* distribution with *k* − 1 df when applicable.

For noncomparative studies reporting proportions, proportional meta-analyses will be conducted using random-effects models. Proportions will be transformed using the Freeman-Tukey double arcsine transformation to stabilize variances and account for the binomial distribution and between-study heterogeneity.

For efficacy and effectiveness outcomes, dichotomous effect measures, including risk ratios (RRs), hazard ratios, odds ratios (ORs), incidence rate ratios, risk differences (RDs), and VE, will be extracted according to the study design and outcome definition. For observational studies, the most fully adjusted effect estimates will be preferentially extracted whenever available. For RCTs, when VE, RRs, or ORs are not reported, RR estimates will be calculated from available data (number of events and total sample size). VE will be used as the preferred summary measure whenever feasible and will be derived from reported effect estimates using standard formulas when not directly reported.

For binary immunogenicity outcomes, comparative studies will be summarized using RRs, ORs, or RDs as appropriate. For noncomparative studies where only proportions are available, results will be synthesized using proportional meta-analysis as described above.

For continuous immunogenicity outcomes, mean differences or standardized mean differences with corresponding 95% CIs (or SEs) will be extracted for comparative studies. For single-arm studies, means or geometric means with their corresponding 95% CIs (or SEs) will be extracted.

Prespecified subgroup analyses will be performed using predefined categories of the following variables: time since vaccination, time from vaccination to delivery, infant age, country income level, geographic region, study design, gestational age at time of vaccination, gestational age at birth, RSV seasonal pattern, infant risk status, maternal risk status, RSV subtype, coadministration with other routinely recommended vaccines in pregnancy, and other maternal risk factors and characteristics.

Heterogeneity will be assessed through visual inspection of forest plots and consideration of the magnitude and direction of effects, CI overlap, and clinical and methodological diversity, as well as using the Cochran Q, *I*^*2*^, and τ^2^. *I*^2^ will not be interpreted using solely fixed thresholds (Table 3).

**Table 3.** Planned subgroup and sensitivity analyses.

Domain	Subgroups and levels^a^	Rationale	Analysis plan
Study design	Type of study design; categories: randomized controlled trials, observational studies (cohort, case-control, test-negative design, cross-sectional studies, or case series), and quasi-experimental studies	The effect of the intervention may differ across specific study designs	Stratification
Gestational age at vaccination	For example, 24‐27.6 wk, 28‐31.6 wk, and 32‐36.6 wk	Antibody transfer increases with longer interval to delivery	Stratified meta-analysis; meta-regression
Time between vaccination and delivery	For example, <14 d, 14-27 d, and ≥28 d	Time-dependent transfer of maternal antibodies to infant	Stratified; continuous meta-regression
Gestational age at birth	Preterm (<37 wk) vs term (≥37 wk)	Preterm infants may receive fewer antibodies	Stratified analysis
Infant risk groups	Comorbidities (eg, CHD^b^ and CLD^c^) and SGA^d^	Differential baseline risk	Stratified analysis
Maternal risk groups	Comorbidities	Differential baseline risk	Stratified analysis
RSV^e^ subtype	RSV-A vs RSV-B	Antigenic differences may affect VE^f^	Stratified analysis
Coadministration	With Tdap, influenza, or COVID-19 vaccines vs none	Immune interference or enhancement	Stratified analysis
Region and income level	World Bank income groups	Epidemiology and exposure differ	Stratified; meta-regression
Risk of bias	Low vs high; adjusted vs unadjusted	Bias can inflate or attenuate effects	Sensitivity exclusions

a Subgroup categories are subject to change based on data availability in the published literature.

b CHD: congenital heart disease.

c CLD: chronic lung disease.

d SGA: small for gestational age.

e RSV: respiratory syncytial virus.

f VE: vaccine efficacy.

Sensitivity analyses will be conducted by excluding studies classified as having a high risk of bias to assess the robustness of the findings.

Potential small-study effects will be assessed using funnel plots and appropriate statistical tests for funnel plot asymmetry (eg, Egger, Harbord, or Peters tests as appropriate) when at least 10 studies are available. Funnel plot asymmetry will be interpreted cautiously as it may reflect publication bias as well as clinical or methodological heterogeneity, selective outcome reporting, or chance [20].

Potential sources of missing data will be assessed, including unreported outcomes, missing summary statistics, and unavailable study-level characteristics. When missing values can be derived from other reported information (eg, CIs, *P* values, or graphical data), they will be calculated using appropriate methods. When missing data cannot be obtained through derivation, analyses will be conducted using the available nonmissing data. All assumptions, derivations, and decisions regarding missing data will be documented to ensure transparency and reproducibility. The final dataset of pooled data will then be summarized and mapped using Microsoft Power BI [21] to create an interactive dashboard for data visualization programmed by the Institute for Clinical Effectiveness and Health Policy team based on this protocol. This platform will allow users to explore maternal and neonatal effectiveness and immunogenicity outcomes through various figures, tables, and maps. Meta-analyses will be performed using R (R Foundation for Statistical Computing) and Shiny (RStudio language) [22], incorporating random-effects models and proportional meta-analyses to synthesize the data based on algorithms that select and automatically calculate the meta-analyses on the Shiny application. Provided that data are available and methodologically suitable, aggregate meta-analyses will be performed for each comparison in accordance with the *Cochrane Handbook for Systematic Reviews of Interventions* using random-effects meta-analysis for primary analyses.

### Certainty of Evidence

We will apply the Grading of Recommendations Assessment, Development, and Evaluation (GRADE) approach [23] to rate certainty for critical outcomes, downgrading for risk of bias, inconsistency, indirectness, imprecision, and publication bias. Summary of findings tables will be prepared for key infant outcomes and maternal outcomes.

### Patient and Public Involvement

No patients will be involved in review development. Dissemination materials will be designed for policymakers and clinicians; lay summaries will be considered as evidence accumulates.

### Ethical Considerations

We will use published and routinely collected anonymized data; ethics approval is not required.

### Dissemination

Findings will be disseminated through peer-reviewed publications and conference presentations and, on a continuous basis, through a publicly accessible interactive dashboard providing real-time meta-analyses 24.

### Amendments

Any protocol amendments will be documented with dates, rationale, and implications for interpretation and reflected in PROSPERO and in a public changelog.

## Results

This living systematic review was funded in June 2025. As of January 2026, a total of 837 records were screened, 105 underwent full-text assessment, and 12 met the inclusion criteria, of which 10 studies contributed extractable data. The evidence base included 1 multicountry randomized trial (with 2 nested substudies) and 9 observational studies, predominantly test-negative case-control designs (Figure 1).

**Figure 1.**
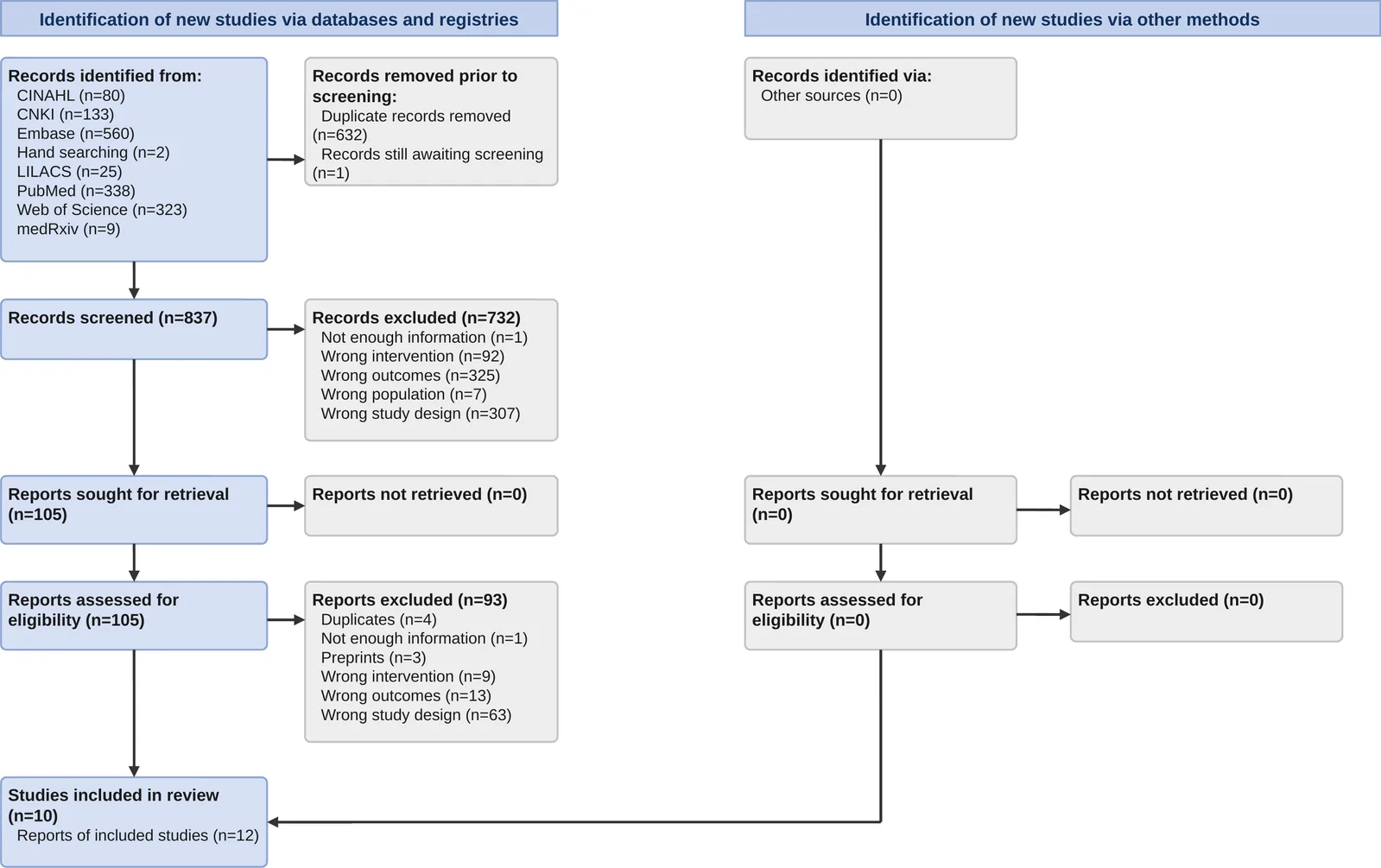
Study flow diagram. CNKI: China National Knowledge Infrastructure.

Of the 10 included studies, 1 was multicountry, 4 were conducted in Argentina, 2 were conducted in the United States, 2 were conducted in the United Kingdom, and 1 was conducted in France. Eight studies assessed VE, 1 assessed both VE and immunogenicity, and 1 assessed immunogenicity only.

Data collection and analysis are ongoing, with monthly updates issued until April 2028 and the first effectiveness estimates available in July 2026.

## Discussion

### Expected Findings

We anticipate that maternal RSVPreF vaccination will be associated with clinically meaningful reductions in RSV-associated disease among infants, particularly RSV hospitalization and severe RSV LRTD, and with a direct protection against RSV illness among pregnant and postpartum individuals. Drawing on evidence from the MATISSE trial and early postlicensure evidence, we expect pooled VE against infant RSV hospitalization to be highest in the first 3 months of life. We further hypothesize that effectiveness will be higher when vaccination occurs at least 14 days before delivery, consistent with the time required for active transplacental maternal immunoglobulin G transfer, and broadly stable across the various gestational age windows during which the vaccine is currently implemented (eg, from 28 to 32 weeks). Immunogenicity outcomes are expected to characterize maternal neutralizing antibody responses and maternal-infant transfer ratios and, where data permit, explore their association with observed effectiveness. These measures are intended to provide mechanistic insights rather than establish clinical protection directly. As this is a living systematic review, these estimates are provisional and will be updated as evidence accumulates.

Previous systematic reviews of maternal RSV vaccination during pregnancy, largely based on randomized trials of different vaccine candidates, have reported VE estimates of approximately 50% to 67% against RSV hospitalization and 74% to 81.9% against severe RSV LRTD in infants under 3 months of age. The present review is intended to complement and extend that body of work in 4 important ways. First, it focuses specifically on the licensed RSVPreF (ABRYSVO) vaccine and evidence generated during the postlicensure real-world period rather than pooling prelicensure trial data. Second, it uses a living review framework with at least monthly surveillance updates available on the online platform, enabling continuous incorporation of rapidly emerging effectiveness data. Third, it prospectively integrates infant and maternal effectiveness and immunogenicity outcomes and prespecifies design-appropriate synthesis methods, including separate pooling of randomized and nonrandomized studies and standardization of adjusted estimates to VE. Fourth, it emphasizes clinically and programmatically actionable relevant subgroups, particularly the timing of vaccination relative to delivery and gestational age at vaccination. Consequently, this review is expected to provide estimates that better reflect vaccine performance under routine programmatic conditions across diverse settings and RSV seasons while also helping characterize the limited evidence regarding the direct benefits of vaccination for pregnant and postpartum individuals.

### Strengths and Limitations

The principal strengths of this review include its exclusive focus on RWE generated under routine programmatic conditions; its living design that enables timely updating in a rapidly evolving field; inclusion of studies from different countries and health system settings; evaluation of clinically meaningful infant and maternal outcomes such as RSV hospitalization, severe RSV LRTD, and intensive care unit admission; prespecified, policy-relevant subgroup analyses; the use of standardized methods for study selection, data extraction, risk-of-bias assessment (RoB 2 and ROBINS-I), and certainty of evidence assessment (GRADE); and the absence of language restrictions, which further reduces the risk of selection bias. The design also has inherent limitations. Postlicensure effectiveness estimates will rely primarily on observational studies, most commonly test-negative and cohort designs, which are susceptible to residual confounding despite statistical adjustment. Variability in outcome definitions and laboratory confirmation methods across studies may introduce outcome misclassification. Early RWE is concentrated in a limited number of high-income countries, potentially restricting generalizability. Several outcomes and subgroups may be informed by only 1 or 2 studies with modest sample sizes and wide CIs, warranting cautious interpretation. Data on preterm infants, protection beyond 6 months of age, and maternal and postpartum outcomes are also likely to remain limited in the near term. Immunogenicity outcomes may be difficult to pool because of heterogeneity in assays and reporting metrics. These limitations will be mitigated through prespecified eligibility criteria, design-stratified random-effects meta-analyses, formal risk-of-bias and certainty (GRADE) assessment, sensitivity analyses excluding studies at high risk of bias, and transparent version control with a publicly accessible changelog.

### Future Research Directions

As the evidence base expands, future iterations of this review will incorporate data from underrepresented settings, including low- and middle-income countries; longer-term follow-up extending beyond 6 months of age; preterm and other high-risk infants; more comprehensive assessments of maternal and postpartum protection; comparisons between maternal vaccination and infant monoclonal antibody prophylaxis; and analyses of antibody durability and potential correlates of protection. Safety outcomes are beyond the scope of this review and will be addressed separately.

### Conclusions

This living systematic review and meta-analysis will provide a continuously updated, methodologically rigorous synthesis of RWE on the effectiveness and immunogenicity of maternal RSVPreF vaccination in infants and pregnant and postpartum individuals. By generating timely, design-appropriate estimates both overall and within policy-relevant subgroups, the review is intended to inform clinical decision-making, maternal immunization programs, and future research priorities as the evidence base continues to evolve.

## Supplementary material

10.2196/94382 Multimedia Appendix 1 Search strategy.

10.2196/94382 Multimedia Appendix 2 Efficacy and effectiveness outcomes and definitions.

10.2196/94382 Checklist 1 PRISMA-P checklist.

## Abbreviations

GRADE: Grading of Recommendations Assessment, Development, and Evaluation
LRTD: lower respiratory tract disease
MATISSE: Maternal Immunization Study for Safety and Efficacy
OR: odds ratio
PRISMA-P: Preferred Reporting Items for Systematic Reviews and Meta-Analyses Protocols
RCT: randomized controlled trial
RD: risk difference
RoB 2: version 2 of the Cochrane risk-of-bias tool for randomized trials
ROBINS-I: Risk of Bias in Nonrandomized Studies of Interventions
RR: risk ratio
RSV: respiratory syncytial virus
RSVPreF: respiratory syncytial virus prefusion F
RWE: real-world evidence
VE: vaccine efficacy
WHO: World Health Organization
