# Supplementary material for: Efficacy, Effectiveness, and Immunogenicity of the Respiratory Syncytial Virus Prefusion F Vaccine Administered During Pregnancy: Protocol for a Living Systematic Review and Meta-Analysis
Source: JMIR Res Protoc. 2026 Oct 15;15:e94382. doi: 10.2196/94382 (PMC13676780; doi:10.2196/94382)
Supplement: Supplementary file 1 [file resprot-v15-e94382-s003.pdf]

PRISMA-P (2015) Checklist – RSVpreF Maternal Vaccination LSR Protocol (JMIRsubmission)

This checklist maps each PRISMA-P item to where it is addressed in the manuscript or its supplements.

Section/Topic	Item	Checklist item	Reported?	Location in manuscript / supplement
Title	1a	Identify the report as a protocol of a systematic review	Yes	Title page; Abstract
Title	1b	If the protocol is for an update of a previous systematic review, identify as such	N/A	—
Registration	2	If registered, provide the name of the registry and registration number	Yes	PROSPERO registration: CRD420251077829; 'PROSPERO registration' section<br>EUPAS Registration: 1000000713
Authors	3a	Provide name, affiliation, and e-mail address of all protocol authors; provide physical mailing address of corresponding author	To be provided at submission	Author details entered in RJMIR submission system
Authors	3b	Describe contributions of protocol authors and identify the guarantor of the review	To be provided at submission	Contributor statement (RJMIR metadata)
Amendments	4	If the protocol represents an amendment of a previously completed or published protocol, identify and list changes	N/A / Future updates	'Amendments' section (future changes will be logged)
Support	5a	Sources of financial or	Yes	'Funding' section

Section/Topic	Item	Checklist item	Reported?	Location in manuscript / supplement
		other support for the review		
Support	5b	Name of sponsor(s)	Yes	'Funding' section
Support	5c	Role of sponsor or funders in protocol development	Yes	'Competing interests' & 'Funding' sections (no role in analysis; investigator-driven)
Introduction	6	Rationale: describe the rationale for the review in the context of what is already known	Yes	Introduction (RSV burden; maternal immunization; only licensed vaccine; need for RWE and LSR)
Introduction	7	Objectives: provide an explicit statement of the question(s) being addressed with reference to participants, interventions, comparators, and outcomes (PICO)	Yes	Objectives; Eligibility criteria (PICOS)
Methods	8	Eligibility criteria (study characteristics and report characteristics)	Yes	'Eligibility criteria (PICOS)'
Methods	9	Information sources (databases with dates of coverage and contact with study authors, trialists, or manufacturers)	Yes	'Information sources and search strategy' (databases, dates, grey literature)
Methods	10	Search strategy: present draft of search strategy for at least one database to be used for the review	Yes (Supplement)	Supplement: Search_Strategy_RSVpreF_LSR_2025-09-10.docx

Section/Topic	Item	Checklist item	Reported?	Location in manuscript / supplement
Study records	11a	Data management: describe the mechanism(s) to manage records and data throughout the review	Yes	'Study records: selection and data management' (REDCap; deduplication; versioning)
Study records	11b	Selection process: state the process for selecting studies (screening, eligibility, inclusion)	Yes	'Study records: selection and data management'
Study records	11c	Data collection process: planned method of extracting data from reports	Yes	'Study records: selection and data management' (piloted REDCap forms; duplicate extraction)
Data items	12	List and define all variables for which data will be sought	Yes	'Study records: selection and data management' (data elements enumerated)
Outcomes and prioritization	13	List and define all outcomes for which data will be sought, including prioritization	Yes	Tables 1–2; 'Eligibility criteria (PICOS)'
Risk of bias	14	Describe anticipated methods for assessing risk of bias of individual studies	Yes	'Risk of bias in individual studies' (RoB 2; ROBINS-I; JBI)
Data synthesis	15a	Describe criteria under which study data will be quantitatively synthesized	Yes	'Data synthesis and statistical analysis'
Data synthesis	15b	If data are appropriate for quantitative synthesis, describe planned summary measures,	Yes	'Data synthesis and statistical analysis'

Section/Topic	Item	Checklist item	Reported?	Location in manuscript / supplement
		methods of handling data, and methods of combining data		
Data synthesis	15c	Describe any proposed additional analyses (e.g., sensitivity, subgroup analyses, meta-regression)	Yes	Table 3; 'Data synthesis and statistical analysis'
Data synthesis	15d	If quantitative synthesis is not appropriate, describe the type of summary planned	Yes	'Data synthesis and statistical analysis' (narrative synthesis for non-combinable data)
Meta-bias(es)	16	Specify any planned assessment of meta-bias(es) (e.g., publication bias, selective reporting)	Yes	'Data synthesis and statistical analysis' (funnel plots; Egger/Begg where ≥10 studies)
Confidence in cumulative evidence	17	Describe how the strength of the body of evidence will be assessed	Yes	'Certainty of evidence' (GRADE; SoF tables)
