# Supplementary material for: Efficacy, Effectiveness, and Immunogenicity of the Respiratory Syncytial Virus Prefusion F Vaccine Administered During Pregnancy: Protocol for a Living Systematic Review and Meta-Analysis
Source: JMIR Res Protoc. 2026 Oct 15;15:e94382. doi: 10.2196/94382 (PMC13676780; doi:10.2196/94382)
Supplement: Supplementary file 2 [file resprot-v15-e94382-s001.docx]

**S1 File: Search strategy**

PubMed 29/5/2025

| Search | Query |
| --- | --- |
| #24 | #8 AND #22 Filters: from 2023/1/1 - 3000/12/12 |
| #23 | #8 AND #22 |
| #22 | #9 OR #10 OR #11 OR #12 OR #13 OR #14 OR #15 OR #16 OR #17 OR #18 OR #19 OR #20 OR #21 |
| #21 | Transplacental[tiab] |
| #20 | Placental[tiab] |
| #19 | Breastmilk[tiab] |
| #18 | Milk[tiab] |
| #17 | Milk, Human[Mesh] |
| #16 | Ante-Natal[tiab] |
| #15 | Antenatal[tiab] |
| #14 | Pre-Natal[tiab] |
| #13 | Prenatal[tiab] |
| #12 | Matern*[tiab] |
| #11 | Pregnan*[tiab] |
| #10 | Pregnant Person[Mesh] |
| #9 | Pregnancy[Mesh] |
| #8 | #1 OR #2 OR #3 OR #4 OR #5 OR #6 OR #7 |
| #7 | Abrysvo[tiab] |
| #6 | PF-06928316[tiab] - Schema: all |
| #5 | Bronchiolitis Vaccin*[tiab] |
| #4 | RSV Vaccin*[tiab] |
| #3 | RSVpreF[tiab] |
| #2 | Syncytial-Virus Vaccin*[tiab] |
| #1 | Respiratory Syncytial Virus Vaccines[Mesh] |

EMBase (Ovid) 29/5/2025

1	exp respiratory syncytial virus vaccine/

2	(Syncytial adj2 Vaccin*).ti,ab.

3	RSVpreF.ti,ab.

4	(RSV adj1 Vaccin*).ti,ab.

5	(Bronchiolitis adj3 Vaccin*).ti,ab.

6	PF-06928316.ti,ab.

7	Abrysvo.ti,ab.

8	or/1-7

9	exp pregnancy/

10	exp pregnant woman/

11	Pregnan*.ti,ab.

12	Matern*.ti,ab.

13	Prenatal.ti,ab.

14	Pre-Natal.ti,ab.

15	Antenatal.ti,ab.

16	Ante-Natal.ti,ab.

17	exp breast milk/

18	Milk.ti,ab.

19	Breastmilk.ti,ab.

20	Placental.ti,ab.

21	Transplacental.ti,ab.

22	or/9-21

23	8 and 22

24	limit 23 to yr="2023 -Current"

Web of Science 29/5/2025

(TS=Respiratory Syncytial Virus Vaccines OR TI=(Syncytial-Virus NEAR/1 Vaccin*) OR AB=(Syncytial-Virus NEAR/1 Vaccin*) OR TI=RSVpreF OR AB=RSVpreF OR TI=(RSV NEAR/1 Vaccin*) OR AB=(RSV NEAR/1 Vaccin*) OR TI=(Bronchiolitis NEAR/3 Vaccin*) OR AB=(Bronchiolitis NEAR/3 Vaccin*) OR TI=PF-06928316 OR AB=PF-06928316 OR TI=Abrysvo OR AB=Abrysvo) AND (TS=Pregnancy OR TS=Pregnant Women OR TI=Pregnan* OR AB=Pregnan* OR TI=Matern* OR AB=Matern* OR TI=Prenatal OR AB=Prenatal OR TI=Pre-Natal OR AB=Pre-Natal OR TI=Antenatal OR AB=Antenatal OR TI=Ante-Natal OR AB=Ante-Natal OR TS=Breast Milk OR TI=Milk OR AB=Milk OR TI=Breastmilk OR AB=Breastmilk OR TI=Placental OR AB=Placental OR TI=Transplacental OR AB=Transplacental)

LILACS 29/5/2025

(MH:(Respiratory Syncytial Virus Vaccines) OR (Syncytial Virus Vaccin*) OR (Vacuna contra el Virus Sincicial) OR (Vacina contra o vírus sincicial) OR (Vacunacion contra el vírus sincicial) OR (Vacinação contra o vírus sincicial) OR (RSVpreF) OR (RSV Vaccin*) OR (Vacuna contra VSR) OR (Vacina contra VSR) OR (Vacunacion contra el VSR) OR (Vacinação contra VSR) OR (Bronchiolitis Vaccin*) OR (Vacuna contra la Bronquiolitis) OR (Vacina contra bronquiolite) OR (Vacunacion contra la bronquiolitis) OR (Vacinação contra bronquiolite) OR (PF-06928316) OR (Abrysvo)) AND (MH:(Pregnancy) OR MH:(Pregnant Person) OR (Pregnan*) OR (Embaraz*) OR (Gravid*) OR (Matern*) OR (Prenatal) OR (Pre-Natal) OR (Antenatal) OR (Ante-Natal) OR MH:(Milk, Human) OR (Milk) OR (Leche) OR (Leite) OR (Breastmilk) OR (Placenta*) OR (Transplacenta*))

CNKI 29/5/2025

(SU=Respiratory Syncytial Virus Vaccines OR TKA=Syncytial-Virus Vaccine OR RSVpreF OR TKA=RSV Vaccine OR TKA=Bronchiolitis Vaccination OR TKA=PF-06928316 OR TKA=Abrysvo) AND (SU=Pregnancy OR SU=Pregnant Women OR TKA=Pregnant OR TKA=Pregnancy OR TKA=Maternal OR TKA=Prenatal OR SU=Breast Human OR TKA=Milk OR TKA=Breastmilk OR TKA=Placental OR TKA=Transplacental)
