# Supplementary material for: Efficacy, Effectiveness, and Immunogenicity of the Respiratory Syncytial Virus Prefusion F Vaccine Administered During Pregnancy: Protocol for a Living Systematic Review and Meta-Analysis
Source: JMIR Res Protoc. 2026 Oct 15;15:e94382. doi: 10.2196/94382 (PMC13676780; doi:10.2196/94382)
Supplement: Supplementary file 3 [file resprot-v15-e94382-s002.docx]

### **Supplementary file 1. Efficacy and effectiveness outcomes and definitions**

| **Outcome** | **Population** | **Variable Operational Definition** |
| --- | --- | --- |
| Any RSV detection (first episode) | Infant | Laboratory-confirmed RSV detection corresponding to the first episode in infants up to 12 months of age. |
| Any RSV detection (any episode) | Infant | Laboratory-confirmed RSV detection corresponding to any episode in infants up to 12 months of age. |
| Any RSV detection (first episode) | Pregnant and postpartum | Laboratory-confirmed RSV detection corresponding to the first episode in pregnant and postpartum individuals (through 12 months post-RSVpreF vaccination) |
| Any RSV detection (any episode) | Pregnant and postpartum | Laboratory-confirmed RSV detection corresponding to any episode in pregnant and postpartum individuals (through 12 months post-RSVpreF vaccination) |
| Asymptomatic RSV infection | Infant | Determined by asymptomatic antibody or antigens (indirect immunofluorescence assay [IFA]; enzyme-linked immunosorbent assay [ELISA]‐based test; direct fluorescent antibody assay [DFA]; Lateral flow immunoassay [LFIA]) and/or asymptomatic RSV infection based on real‐time Polymerase Chain Reaction (RT-PCR) (LNA one‐tube nested real‐time [OTNRT]‐PCR, rapid reverse‐transcription recombinase‐aided amplification [RT‐RAA] assay; reverse transcription strand invasion‐based amplification [RT‐SIBA]) in infants up to 12 months of age |
| Asymptomatic RSV infection | Pregnant and postpartum | Determined by asymptomatic antibody or antigens (IFA; ELISA‐based test; DFA; LFIA) and/or asymptomatic RSV infection based on RT-PCR (LNA OTNRT‐PCR, RT‐RAA assay; RT‐SIBA) in pregnant and postpartum individuals (through 12 months post-RSVpreF vaccination) |
| RSV hospitalization (first episode) | Infant | First hospitalization due to laboratory-confirmed RSV illness in infants up to 12 months of age. |
| RSV hospitalization  (first episode) | Pregnant and postpartum | First hospitalization due to laboratory-confirmed RSV illness in pregnant and postpartum individuals (through 12 months post-RSVpreF vaccination) |
| RSV hospitalization (any episode) | Infant | Any hospitalization due to laboratory-confirmed RSV illness in infants up to 12 months of age. |
| RSV hospitalization  (any episode) | Pregnant and postpartum | Any hospitalization due to laboratory-confirmed RSV illness in pregnant and postpartum individuals (through 12 months post-RSVpreF vaccination) |
| RSV complication: Secondary bacterial infection (first episode) | Infant | First occurrence of secondary bacterial infection following RSV ARI/LRTI/LRTD in infants up to 12 months of age. |
| RSV complication: Secondary bacterial infection (any episode) | Infant | Any occurrence of secondary bacterial infection following RSV ARI/LRTI/LRTD in infants up to 12 months of age. |
| RSV complication: Respiratory failure (first episode) | Infant | First occurrence of respiratory failure following RSV ARI/LRTI/LRTD in infants up to 12 months of age. |
| RSV complication: Respiratory failure (any episode) | Infant | Any occurrence of respiratory failure following RSV ARI/LRTI/LRTD in infants up to 12 months of age. |
| RSV complication: Multiorgan failure (first episode) | Infant | First occurrence of multiorgan failure following RSV ARI/LRTI/LRTD in infants up to 12 months of age. |
| RSV complication: Multiorgan failure (any episode) | Infant | Any occurrence of multiorgan failure following RSV ARI/LRTI/LRTD in infants up to 12 months of age. |
| RSV complication: Death | Infant | Death following RSV ARI/LRTI/LRTD in infants up to 12 months of age. |
| RSV ARI (first episode) | Infants | First occurrence of clinical disease consistent with RSV ARI (based on lab confirmed RSV testing and/or relevant diagnostic codes) in infants up to 12 months of age |
| RSV ARI (any episode) | Infants | Any occurrence of clinical disease consistent with RSV ARI (based on lab confirmed RSV testing and/or relevant diagnostic codes) in infants up to 12 months of age |
| RSV ARI (first episode) | Pregnant and postpartum | First occurrence of clinical disease consistent with RSV ARI (based on lab confirmed RSV testing and/or relevant diagnostic codes) in pregnant and postpartum individuals (through 12 months post-RSVpreF vaccination) |
| RSV ARI (any episode) | Pregnant and postpartum | Any occurrence of clinical disease consistent with RSV ARI (based on lab confirmed RSV testing and/or relevant diagnostic codes) in pregnant and postpartum individuals (through 12 months post-RSVpreF vaccination) |
| RSV LRTI/LRTD (first episode) | Infant | First occurrence of clinical disease consistent with RSV LRTI/LRTD (based on lab confirmed RSV testing and/or relevant diagnostic codes) in infants up to 12 months of age |
| RSV LRTI/LRTD (any episode) | Infant | Any occurrence of clinical disease consistent with RSV LRTI/LRTD (based on lab confirmed RSV testing and/or relevant diagnostic codes) in infants up to 12 months of age |
| RSV LRTI/LRTD (first episode) | Pregnant and postpartum | First occurrence of clinical disease consistent with RSV LRTI/LRTD (based on lab confirmed RSV testing and/or relevant diagnostic codes) in pregnant and postpartum individuals (through 12 months post-RSVpreF vaccination) |
| RSV LRTI/LRTD (any episode) | Pregnant and postpartum | Any occurrence of clinical disease consistent with RSV LRTI/LRTD (based on lab confirmed RSV testing and/or relevant diagnostic codes) in pregnant and postpartum individuals (through 12 months post-RSVpreF vaccination) |
| RSV LRTI/LRTD hospitalization (first episode) | Infant | First hospitalization due to clinical disease consistent with RSV LRTI/LRTD (based on lab confirmed RSV testing and/or relevant diagnostic codes) in infants up to 12 months of age |
| RSV LRTI/LRTD hospitalization (any episode) | Infant | Any hospitalization due to clinical disease consistent with RSV LRTI/LRTD (based on lab confirmed RSV testing and/or relevant diagnostic codes) in infants up to 12 months of age |
| RSV LRTI/LRTD hospitalization (first episode) | Pregnant and postpartum | First hospitalization due to clinical disease consistent with RSV LRTI/LRTD (based on lab confirmed RSV testing and/or relevant diagnostic codes) in pregnant and postpartum individuals (through 12 months post-RSVpreF vaccination) |
| RSV LRTI/LRTD hospitalization (any episode) | Pregnant and postpartum | Any hospitalization due to clinical disease consistent with RSV LRTI/LRTD (based on lab confirmed RSV testing and/or relevant diagnostic codes) in pregnant and postpartum individuals (through 12 months post-RSVpreF vaccination) |
| All cause LRTI/LRTD (first episode) | Infant | First occurrence of clinical disease consistent with LRTI/LRTD in infants up to 12 months of age |
| All cause LRTI/LRTD (any episode) | Infant | Any occurrence of clinical disease consistent with LRTI/LRTD in infants up to 12 months of age |
| All cause LRTI/LRTD (first episode) | Pregnant and postpartum | First occurrence of clinical disease consistent with LRTI/LRTD in pregnant and postpartum individuals (through 12 months post-RSVpreF vaccination) |
| All cause LRTI/LRTD (any episode) | Pregnant and postpartum | Any occurrence of clinical disease consistent with LRTI/LRTD in pregnant and postpartum individuals (through 12 months post-RSVpreF vaccination) |
| All-cause LRTI/LRTD hospitalization (first episode) | Infant | First hospitalization due to clinical disease consistent with LRTI/LRTD in infants up to 12 months of age |
| All-cause LRTI/LRTD hospitalization (any episode) | Infant | Any hospitalization due to clinical disease consistent with LRTI/LRTD in infants up to 12 months of age |
| All-cause LRTI/LRTD hospitalization (first episode) | Pregnant and postpartum | First hospitalization due to clinical disease consistent with LRTI/LRTD in pregnant and postpartum individuals (through 12 months post-RSVpreF vaccination) |
| All-cause LRTI/LRTD hospitalization (any episode) | Pregnant and postpartum | Any hospitalization due to clinical disease consistent with LRTI/LRTD in pregnant and postpartum individuals (through 12 months post-RSVpreF vaccination) |
| All cause ARI (first episode) | Infant | First occurrence of clinical disease consistent with ARI in infants up to 12 months of age |
| All cause ARI (any episode) | Infant | Any occurrence of clinical disease consistent with ARI in infants up to 12 months of age |
| All cause ARI (first episode) | Pregnant and postpartum | First occurrence of clinical disease consistent with ARI in pregnant and postpartum individuals (through 12 months post-RSVpreF vaccination) |
| All cause ARI (any episode) | Pregnant and postpartum | Any occurrence of clinical disease consistent with ARI in pregnant and postpartum individuals (through 12 months post-RSVpreF vaccination) |
| Antibiotic use in infants with RSV infection (from birth through 12 months of age). | Infant | Antibiotic use in infants with RSV infection from birth through 12 months of age |
